# Relationship between intraoperative hypothermia and hyperthermia with postoperative pulmonary infection and surgical site infection in major non-cardiac surgery

**DOI:** 10.3389/fmed.2024.1408342

**Published:** 2024-08-12

**Authors:** Qian-Yun Pang, Ya-Jun Yang, Yu-Mei Feng, Shu-Fang Sun, Hong-Liang Liu

**Affiliations:** Department of Anesthesiology, Chongqing University Cancer Hospital, Chongqing, China

**Keywords:** hypothermia, hyperthermia, pulmonary infection, surgical site infection, major non-cardiac surgery

## Abstract

**Background:**

Surgical patients often experience intraoperative hypothermia or hyperthermia. However, the relationship of intraoperative hypothermia and hyperthermia with postoperative pulmonary infection (PPI) and surgical site infection (SSI) is unclear. Here, we conducted a retrospective cohort study to address these issues.

**Methods:**

Adult patients who underwent major non-cardiac surgery under general anesthesia were eligible for the study and were recruited. Three indices of core body temperature under hypothermia (<36°C) and hyperthermia (>37.3°C) were calculated as mentioned in the following: absolute value (^0^C), duration of exposure (min), and area under the curve (AUC,°C× min). The outcomes were in-hospital PPI and SSI. The risk-adjusted association of intraoperative hypothermia and hyperthermia with PPI and SSI was determined.

**Results:**

The absolute value (the nadir value of hypothermia and the peak value of hyperthermia) was not associated with PPI and SSI. PPI was associated with (1) duration: hypothermia >90 min [adjusted odds ratio (aOR): 1.425, 95% confidence interval (CI): 1.131–1.796] and hyperthermia >75 min (aOR: 1.395, 95%CI: 1.208–1.612) and (2) AUC: hypothermia >3,198 (aOR: 1.390, 95%CI: 1.128–1.731) and hyperthermia >7,945 (aOR: 2.045, 95%CI: 1.138–3.676). SSI was associated with (1) duration: hypothermia > 195 min (aOR: 2.900, 95%CI: 1.703–4.937) and hyperthermia >75 min (aOR: 1.395, 95%CI: 1.208–1.612) and (2) AUC: hypothermia >6,946 (aOR: 2.665, 95%CI: 1.618–4.390), hyperthermia >7,945 (aOR: 2.619, 95%CI: 1.625–4.220). Interactions were not observed between hyperthermia and hypothermia on the outcomes.

**Conclusions:**

It was observed that intraoperative hypothermia and hyperthermia are associated with postoperative pulmonary infection and surgical site infection in major non-cardiac surgery.

## Introduction

Surgical patients often experience intraoperative hypothermia (core body temperature <36.0°C) (1), which is a very common event during non-cardiac surgery with an incidence of 50%−90% (2–4). Many studies have shown that it is associated with postoperative infectious complications (3, 5–7), whereas other studies have revealed contradictory results (2, 4, 8, 9). Patients might develop intraoperative hyperthermia as well. A recent study reported that hyperthermia at the time of admission to the hospital was associated with mortality in septic patients (10). As hyperthermia has not been well defined, the relationship between intraoperative hyperthermia and postoperative infections remains unclear.

Surgical site infection (SSI) and postoperative pulmonary infection (PPI) are the most common infectious complications, with incidences of 0.7%−33% and 2%−3% in non-cardiac surgery (5, 11). In the past years, much attention has been focused on the prevention of hypothermia, whereas hyperthermia has received little attention. A crucial issue that needs to be addressed is determining, how long is too long and how low or high is too low or high for hypothermia or hyperthermia to increase the risk of PPI or SSI when hypothermia or hyperthermia develops.

Therefore, we conducted a retrospective cohort study to investigate the relationship of intraoperative hypothermia and hyperthermia with PPI and SSI in adult patients undergoing major non-cardiac surgery.

## Methods

### Study design and participants

This retrospective observational study was approved by the ethics committee of Chongqing University Cancer Hospital (No. CZLS2024020-A) and adhered to STROBE guidelines. The STORBE checklist is attached as Supplementary material 1. As this was a retrospective study on a large number of patients, the data were anonymized, and the need for informed consent from patients was exempted.

The participants included patients who underwent major non-cardiac surgeries between Jan 2017 and Dec 2022 in Chongqing University Cancer Hospital. The inclusion criteria were as follows: (1) age > 18 years; (2) general anesthesia; (3) major non-cardiac surgery, which referred to the surgical procedure associated with significant fluid shifts that required postoperative hospitalization, including open resection of organs, thoracic surgery, intracranial surgery, spinal surgery, large joint replacement, and laparoscopic surgery (12); (4) core temperature monitoring (the site at the nasopharynx or oropharynx was routinely used in our institution); and (5) the data of the first major non-cardiac surgery were retrieved when multiple major surgeries were performed in one patient during the study period. The exclusion criteria were as follows: (1) a less than 30 min accumulative duration of core body temperature monitoring, (2) peripheral skin temperature monitoring, (3) a history of long-term steroids intake, (4) systemic infectious disease or pulmonary infection within 1 month before surgery, and (5) missing temperature monitoring or outcome data.

### The data collection

The data of all patients who met the inclusion criteria were retrieved from the electronic medical record system of Chongqing University Cancer Hospital. The data included information about the participants' characteristics [age, sex, body mass index (BMI), American society of anesthesiologists classification physical status (ASA PS), and comorbidities, such as heart disease, hypertension, diabetes mellitus (DM), renal disease, liver disease, arrhythmia, stroke, chronic obstructive pulmonary disease (COPD), thyroid disease, and smoking], preoperative long-term medications (antihypertensive agents, antiplatelets, anticoagulants, and hypoglycemic agents), preoperative laboratory tests [hemoglobin (Hb), glucose, albumin (ALB), and white blood cells (WBCs)], and intraoperative data (surgical duration, bleeding, transfusion, infusion volume, hypothermia, and hyperthermia).

### Temperature monitoring method

Core body temperature was continuously monitored using the temperature probe connected to the anesthesia monitor (BeneView T5, Mindray). The temperature probe was placed in the oropharynx or nasopharynx but not in the urinary tract or anus in our institution.

### Core temperature

Intraoperative core body temperature was collected electronically. Hypothermia was defined as core body temperature below 36°C, and hyperthermia was defined as core body temperature above 37.3°C based on a large data cohort study, in which the mean baseline body temperature of patients was 36.6°C, with a 95% range of 35.3–37.3°C (13). Therefore, 36.0–37.3°C was set as the normal range (reference) in this study. To quantitatively assess the degree of hypothermia and hyperthermia, the following three indices were calculated: (1) the nadir temperature below 36.0°C and the peak temperature above 37.3°C, (2) the accumulative duration (min) below 36°C and above 37.3°C, and (3) the accumulative area under the curve below 36°C and above 37.3°C (AUC, value × exposure, unit: °C × min), which represented the severity of hypothermia and hyperthermia, respectively.

### The outcomes

The outcomes were in-hospital PPI (ICD-10 code: J98.402) and SSI (ICD-10 code: T81.402). SSI was classified as superficial (skin and subcutaneous tissues only), deep (deeper soft tissues, such as fascia and muscle layers), and organ space (any part of the anatomy that was opened or manipulated during surgery). All diagnoses of PPI and SSI were checked and confirmed according to the criteria from the Centers for Disease Control and Prevention of the United States (14, 15).

### Sample size

The available sample size was 37,388 patients. Given a type I error rate of 5% and a background incidence of PPI and SSI of 4.5%, we had 99% power to detect a 0.5% difference between groups. A minimum sample size of 32,000 patients was required to detect the difference.

### Statistical analysis

Continuous variables were presented as median (interquartile range), where those with normal distribution were compared using a *t*-test and those with non-normal distribution were compared using a Mann–Whitney U test. Categorized variables were presented as *n*(%) and compared using Fisher's exact test.

A restricted cubic spline analysis was conducted, and the dose- response relationship was evaluated between the absolute value, duration, and AUC and PPI and SSI. The adjusted odds ratios (aORs) and 95% confidence interval (CI) were calculated using logistic regression. The confounding variables for the adjustment were age, sex, ASA PS scores, BMI, smoking, preoperative glucose, preoperative albumin, preoperative WBCs, preoperative hemoglobin, surgical duration, transfusion, bleeding, and infusion volume. Three knots from the restricted cubic spline analysis were set and located at the 10^th^, 50^th^, and 90^th^ percentiles. The indices of body temperature were categorized into quartiles. Multivariate logistic regression models were used to calculate the aORs and 95%CI in the quartiles of the indices of hypothermia and hyperthermia for the risk of SSI and PPI compared with the normal range of body temperature, and the adjusted confounding variables were as above mentioned.

Patients with missing intraoperative body temperature or outcome data were excluded from the final analysis. Other variables had missing values of <5%, and we imputed the median value.

As an individual might develop both hypothermia and hyperthermia during surgery, the interactions were tested by analyzing the joint effects of intraoperative hypothermia and hyperthermia to explore whether the risk of PPI and SSI for exposure to intraoperative hypothermia was influenced by intraoperative hyperthermia.

In a *post-hoc* sensitivity analysis, we found that patients who were admitted to the ICU after surgery had a higher risk of PPI and SSI; hence, we eliminated the patients admitted to the ICU and repeated the primary analysis.

Stata 16 (Stata Corp LP, USA) and R software 4.12 (R foundation for statistical computing) were used for data analyses. The significance of all tests was two-sided, and a *p-*value of <0.05 was considered statistically significant.

## Results

**Figure 1:**
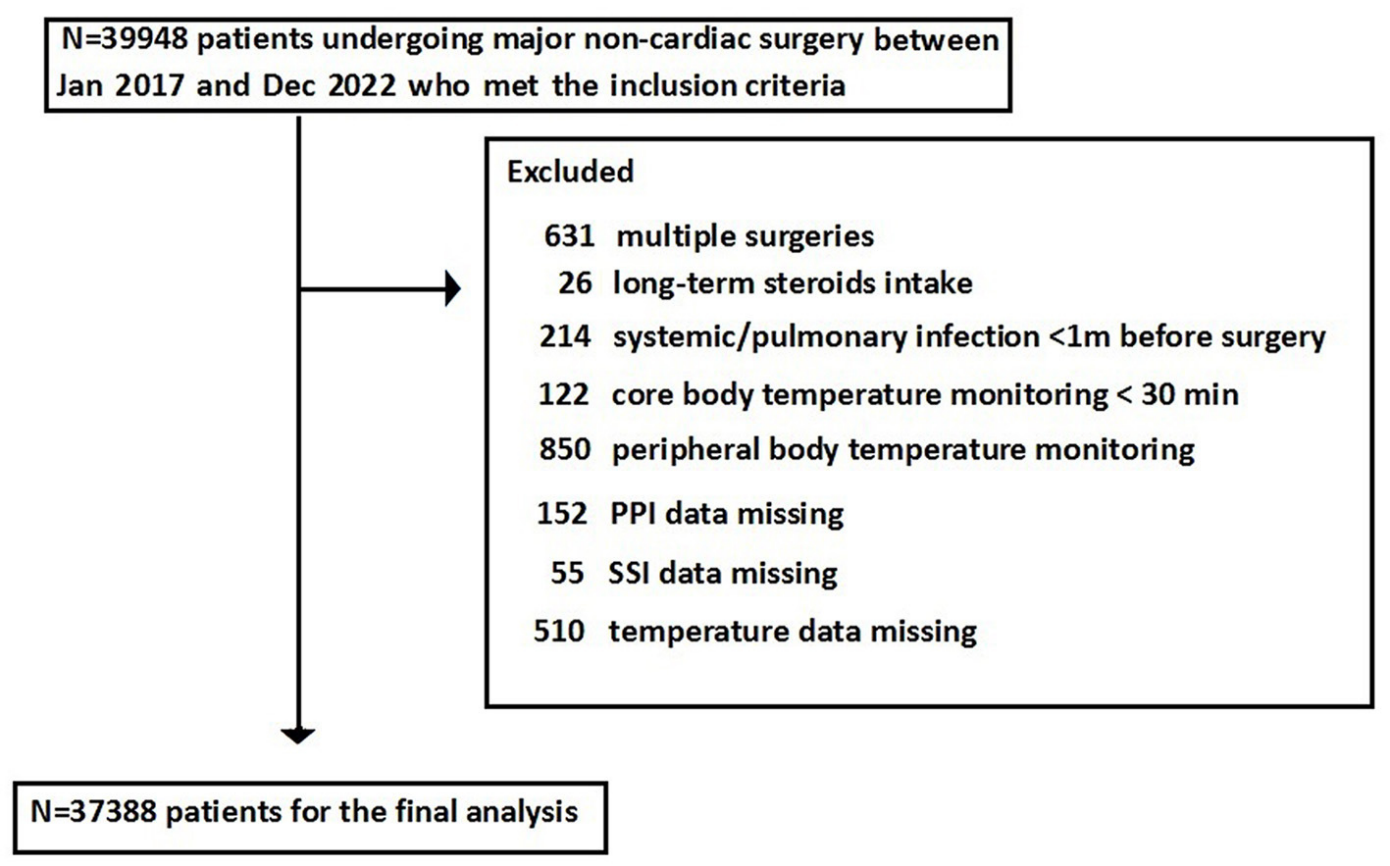
A flow chart depicting the screening of patients.

**Figure 2:**
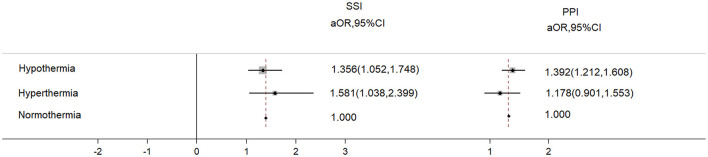
The adjusted odds ratio of PPI and SSI from hypothermia and hyperthermia. The adjusted variables were age, sex, ASA scores, BMI, smoking, preoperative glucose, preoperative albumin, preoperative WBC, preoperative hemoglobin, surgical duration, transfusion, bleeding, and infusion volume.

A total of 39,948 patients met the inclusion criteria and were included in the study; however, 2,560 patients were excluded as they could not meet the exclusion criteria, leaving 37,388 patients for the final analysis (Figure 1). The overall incidence of PPI was 4.6%, and that of SSI was 1.1%. Patients who developed PPI or SSI were more likely to experience hypothermia or hyperthermia and had higher ASA PS scores and older ages compared with those without PPI or SSI (Table 1). The cohort included 29,932 patients with normal body temperature (reference). A total of 6,174 patients with hypothermia had a higher risk of SSI (aOR: 1.356, 95%CI: 1.052–1.748) and PPI (aOR: 1.392, 95%CI: 1.212–1.608), and 1,384 patients with hyperthermia had a higher risk of SSI (aOR: 1.581, 95%CI: 1.038–2.399) but not PPI (aOR: 1.178, 95%CI: 0.901–1.553) (Figure 2).

**Table 1:** The characteristics of patients with and without SSI and with and without PPI.

**Variable**	**PPI (*n =* 1,326)**	**Non-PPI (*n =* 36,062)**	***p*-value**	**SSI (*n =* 413)**	**Non-SSI (*n =* 36,975)**	***p*-value**
Age (y)	62 (53,68)	53 (46,62)	0.000	58 (51,67)	53 (46,63)	0.000
Sex			0.000			0.000
Male	820 (6.39%)	12,021 (93.61%)		233	12,608	
Female	506 (2.06%)	24,041 (97.94%)		180	24,367	
BMI (kg/cm^2^)	23.1 (21.4,25.4)	23.5 (21.5,25.7)	0.000	23.4 (21.3,25.4)	23.5 (21.4,25.7)	0.135
ASA PS			0.000			0.000
I	3 (0.79%)	379 (1.05%)		1 (0.24%)	381 (1.03%)	
II	386 (29.11%)	21,212 (58.82%)		152 (36.80%)	21,446 (58.00%)	
III	883 (66.59%)	14,085 (39.06%)		244 (59.08%)	14,724 (39.82%)	
IV	52 (3.92%)	376 (1.04%)		16 (3.87%)	412 (1.11%)	
V	2 (0.03%)	10 (0.03%)		0 (0%)	12 (0.03%)	
**Comorbidity**						
Heart disease	56 (6.97%)	1,270 (3.47%)	0.000	14 (1.74%)	399 (1.09%)	0.080
Hypertension	275 (4.44%)	1,051 (3.37%)	0.000	73 (1.18%)	340 (1.09%)	0.538
DM	119 (4.83%)	1,207 (3.46%)	0.000	33 (1.34%)	380 (1.09%)	0.251
Renal disease	13 (10.24%)	1,313 (3.52%)	0.000	2 (1.57%)	411 (1.10%)	0.612
Liver disease	22 (2.57%)	1,304 (3.57%)	0.118	13 (1.52%)	400 (1.09%)	0.241
Arrhythmia	103 (5.14%)	1,223 (3.46%)	0.000	30 (1.5%)	383 (1.08%)	0.083
Stroke	51 (8.12%)	1,275 (3.47%)	0.000	8 (1.27%)	405 (1.10%)	0.682
COPD	12 (6.78%)	1,314 (3.53%)	0.020	4 (2.26%)	409 (1.1%)	0.140
Thyroid disease	17 (3.25%)	1,309 (3.55%)	0.712	5 (0.96%)	408 (1.11%)	0.743
Smoking	342 (7.12%)	984 (3.02%)	0.000	14 (1.74%)	399 (1.09%)	0.682
**Preoperative medication**						
Antihypertension	136 (4.52%)	1,190 (3.46%)	0.003	36 (1.2%)	377 (1.1%)	0.257
Antiplatelets	16 (5.02%)	1,310 (3.53%)	0.154	4 (1.25%)	409 (1.1%)	0.798
Anticoagulants	4 (7.84%)	1,322 (3.54%)	0.097	0 (0%)	413 (1.11%)	0.450
Hypoglycemic	77 (4.9%)	1,249 (3.49%)	0.003	21 (1.34%)	392 (0.09%)	0.372
**Preoperative tests**						
Hb (g/L)	127 (122,137)	127 (121,137)	0.364	127 (114,135)	127 (121,137)	0.000
Glucose (mmol/L)	5.10 (4.43,5.35)	5.02 (4.48,5.26)	0.038	5.1 (4.5,5.5)	5.0 (4.5,5.3)	0.098
ALB (g/L)	42.0 (38.2,42.0)	42.0 (39.8,44.4)	0.000	42 (38.3,42.6)	42 (39.8,44.3)	0.000
WBC (^*^10^9^/L)	6.15 (5.13,6.93)	6.15 (4.97,6.67)	0.000	6.15 (5.03,6.81)	6.15 (4.98,6.68)	0.675
Surgical duration (min)	190 (133,275)	135 (95,204)	0.000	220 (145,300)	135 (95,205)	0.000
Bleeding (ml)	100 (50,200)	100 (50,100)	0.000	100 (50,150)	100 (50,100)	0.323
Transfusion	40 (3.02%)	680 (1.89%)	0.003	15 (2.08%)	398 (1.09%)	0.011
Fluid infusion (ml)	2,200 (1,500, 3,000)	1,600 (1,100, 2,300)	0.000	2,400 (1,600, 3,350)	1,600 (1,200, 2,300)	0.000
Hypothermia	289 (21.79%)	5,884 (16.32%)	0.000	80 (19.37%)	6,093 (16.48%)	0.115
Hyperthermia	66 (4.98%)	1,318 (3.65%)	0.012	27 (6.54%)	1,357 (3.67%)	0.002

SSI, surgical site infection; PPI, postoperative pulmonary infection; BMI, body mass index; ASA PS, American society of anesthesiologists classification physical status; DM, diabetes mellitus; COPD, chronic obstructive pulmonary disease; Hb, hemoglobin; ALB, albumin; WBC, white blood cell.

When the indices of body temperature were considered continuous variables, the nadir value of hypothermia with each 0.1°C decrease was not associated with SSI or PPI. However, the duration of hypothermia with each minute increase increased the odds of SSI (aOR: 1.003, 95%CI: 1.001–1.004) and PPI (aOR: 1.002, 95%CI: 1.001–1.003), and the AUC of hypothermia with each °C × min increase increased the odds of SSI (aOR: 1.000, 95%CI: 1.000–1.000) and PPI (aOR: 1.000, 95%CI: 1.000–1.000). The peak value of hyperthermia with each 0.1°C increase or the duration of hyperthermia with each minute increase was not associated with SSI or PPI, but the AUC with each °C × min increase slightly increased the risk of PPI (aOR: 1.000, 95%CI: 1.000–1.000) (Table 2).

**Table 2:** The relationship between the indices (as continuous variables) and SSI and PPI.

	**PPI**			**SSI**		
	**aOR**	**95%CI**	***p*** **-value**	**aOR**	**95%CI**	**p-value**
**Hypothermia**						
Nadir value (each 0.1°C decrease)	1.328	0.766–2.305	0.313	1.100	0.427–2.836	0.843
Duration (each min increase)	1.002	1.001–1.003	0.000	1.003	1.001–1.004	0.000
AUC (each °C^*^min increase)	1.000	1.000–1.000	0.000	1.000	1.000–1.000	0.000
**Hyperthermia**						
Peak value (each 0.1°C increase)	0.759	0.355–1.623	0.476	0.959	0.318–2.894	0.941
Duration (each min increase)	1.046	0.804–1.361	0.467	1.003	0.999–1.005	0.061
AUC (each °C^*^min increase)	1.000	1.000–1.000	0.037	1.000	0.999–1.000	0.056

SSI, surgical site infection; PPI, postoperative pulmonary infection; Nadir value, the lowest value under hypothermia; AUC, area under the curve; Peak value, the highest value under hyperthermia.

As shown in Table 3, the indices of body temperature were categorized into quartiles, and the normal temperature range (36.0–37.3°C) was set as the reference. Multivariable regression analysis revealed that the nadir value of hypothermia from the 1^st^ quartile to the 4^th^ quartile was not associated with the risk of PPI and SSI. There was a gradient increasing risk of PPI or SSI from the 1^st^ to the 4^th^ quartile of the duration or AUC of hypothermia. The threshold of duration for increasing the risk of PPI was 90 min (aOR: 1.425, 95%CI: 1.131–1.796), while the threshold of duration for increasing the risk of SSI was 195 min (aOR: 2.900, 95%CI: 1.703–4.937). The odds of PPI increased in the 3^rd^ and 4^th^ quartiles of AUC of hypothermia (aOR: 1.390, 95%CI: 1.128–1.731 and aOR: 1.931, 95%CI: 1.381–2.700), and the odds of SSI increased in the 4^th^ quartile (aOR: 2.665, 95%CI: 1.618–4.390). The odds ratio of PPI or SSI was not increased from the 1^st^ to the 4^th^ quartile of the peak value of hyperthermia. The threshold of duration for increasing the risk of PPI and SSI was 75 min (aOR: 1.395, 95%CI: 1.208–1.612 and aOR: 2.962, 95%CI: 1.833–4.785). The odds ratio of PPI and SSI was increased in the 4^th^ quartile of the AUC of hyperthermia (aOR: 2.045, 95%CI: 1.138–3.676 and aOR: 2.619, 95%CI: 1.625–4.220).

**Table 3:** The risk of PPI and SSI in the categorized quartiles of the indices of hypothermia and hyperthermia compared with the reference.

	**PPI**		**SSI**	
	**aOR (95%CI)**	***p*** **-value**	**aOR (95%CI)**	***p*** **-value**
**Hypothermia**				
**Nadir value**				
Q1 (<35.3°C) (*n =* 439)	0.986 (0.755,1.288)	0.918	0.554 (0.177,1.734)	0.310
Q2 (35.3–35.7°C) (*n =* 1664)	0.894 (0.701,1.140)	0.368	0.758 (0.443,1.297)	0.312
Q3 (35.7–35.9°C) (*n =* 2317)	0.996 (0.666,1.179)	0.407	0.847 (0.549,1.307)	0.454
Q4 (≥35.9°C) (*n =* 1753)	0.712 (0.399,1.271)	0.252	0.925 (0.574,1.490)	0.749
**Duration**				
Q1 (0–30 min) (*n =* 1830)	1.046 (0.804,1.361)	0.738	0.883(0.532,1.466)	0.630
Q2 (30–90 min) (*n =* 2230)	1.163 (0.925,1.461)	0.196	0.898(0.569,1.417)	0.644
Q3 (90–195 min) (*n =* 1682)	1.425 (1.131,1.796)	0.003	1.394 (0.922,2.108)	0.115
Q4 (>195 min) (*n =* 431)	1.932 (1.343,2.778)	0.000	2.900(1.703,4.937)	0.000
**AUC**				
Q1 (<1,077) (*n =* 1,412)	0.948 (0.706,1.273)	0.725	0.618 (0.329,1.163)	0.136
Q2 (1,077–3,198) (*n =* 2,227)	1.207 (1.128,1.713)	0.090	1.011 (0.671,1.522)	0.959
Q3 (3,198–6,946) (*n =* 2,022)	1.390 (1.128,1.713)	0.002	1.182 (0.901,1.746)	0.399
Q4 (>6,946) (*n =* 512)	1.931 (1.381,2.700)	0.000	2.665 (1.618,4.390)	0.000
**Hyperthermia**				
**Peak value**				
Q1 (=37.3°C) (*n =* 402)	1.298 (0.829,2.031)	0.254	0.391 (0.097,1.575)	0.187
Q2 (37.3–37.5°C) (*n =* 476)	0.940 (0.575,1.537)	0.806	0.879 (0.361,2.137)	0.776
Q3 (37.5–38°C) (*n =* 387)	0.732 (0.399,1.342)	0.313	0.424 (0.105,1.710)	0.228
Q4 (>38°C) (*n =* 119)	1.235 (0.499,3.056)	0.649	1(omitted)	
**Duration**				
Q1 (0–15 min) (*n =* 191)	1.034 (0.671,1.593)	0.879	0.590 (0.082,4.252)	0.601
Q2 (15–75 min) (*n =* 561)	1.137 (0.789,1.638)	0.492	0.782 (0.366,1.671)	0.526
Q3 (75–210 min) (*n =* 501)	1.395 (1.208,1.612)	0.000	2.962 (1.833,4.785)	0.000
Q4 (>210 min) (*n =* 131)	2.017 (1.145,3.877)	0.017	1.356 (1.048,1.754)	0.021
**AUC**				
Q1 (<560) (*n =* 151)	1.538 (0.746,3.171)	0.244	1.868 (0.591,5.906)	0.287
Q2 (560–2,803) (*n =* 535)	0.935 (0.591,1.478)	0.773	0.593 (0.218,1.608)	0.304
Q3 (2803–7945) (*n =* 559)	1.000 (0.661,1.514)	0.998	0.521 (0.072,3.748)	0.517
Q4 (>7,945) (*n =* 139)	2.045 (1.138,3.676)	0.017	2.619 (1.625,4.220)	0.000

PPI, postoperative pulmonary infection; SSI, surgical site infection; Q1, the 1st quartile; Q2, the 2nd quartile; Q3, the 3rd quartile; Q4, the 4th quartile. Nadir value, the lowest value under hypothermia; Peak value, the highest value under hyperthermia; AUC, area under the curve.

An interaction analysis was conducted to explore whether the risk of PPI and SSI under intraoperative hypothermia was influenced by intraoperative hyperthermia (Table 4). The results showed that there were no interactions between hypothermia and hyperthermia either in the absolute value or in the duration or AUC.

**Table 4:** The interaction analysis between hypothermia and hyperthermia.

	**aORs (95%CI)**	**p-value**
**PPI**		
Peak value(hyperthermia)^*^nadir value (hypothermia)	0.891(0.659,1.204)	0.452
Duration of hyperthermia^*^duration of hypothermia	1.000(1.000,1.000)	0.803
AUC of hyperthermia^*^AUC of hypothermia	0.968(0.843,1.112)	0.648
**SSI**		
Peak value(hyperthermia)^*^nadir value (hypothermia)	1.404 (0.319,6.170)	0.653
Duration of hyperthermia^*^duration of hypothermia	1.000(1.000,1.000)	0.846
AUC of hyperthermia^*^AUC of hypothermia	0.972(0.784,1.206)	0.799

PPI, postoperative pulmonary infection; SSI, surgical site infection.

A *post-hoc* sensitivity analysis was conducted when eliminating patients admitted to the ICU after surgery. The results showed that the characteristics were not different between patients admitted to the ICU and those not admitted to the ICU (Supplementary Table 1). When excluding patients without ICU admission from the analysis, the nadir value, duration, or AUC of hypothermia in the quartiles did not increase the odds of PPI or SSI. The peak value or duration of hyperthermia in the quartiles did not increase the risk of PPI or SSI, whereas the AUC of hyperthermia in the 3^rd^ quartile slightly increased the risk of PPI (aOR: 1.685, 95%CI: 1.013 to 2.801) (Supplementary Table 2). These results indicated that when patients without ICU admission were excluded from the analysis, hypothermia was not associated with PPI or SSI and that the relationship between PPI and hyperthermia was not changed.

## Discussion

The results of our study showed that intraoperative hypothermia and hyperthermia increased the odds of PPI and SSI and indicated that the duration and AUC of hypothermia and hyperthermia could predict PPI and SSI.

In the past years, much attention has been focused on the consequences and prevention of intraoperative hypothermia, but the studies revealed contradictory results concerning the relationship between hypothermia and infectious outcomes (2–9). The reasons might be that the majority of studies used only the absolute value of body temperature and the criteria for hypothermia were not consistent among these studies. In our study, we categorized core body temperature into three indices (absolute value, duration, and AUC) and found that the duration and AUC of hypothermia and hyperthermia could predict PPI and SSI but the absolute value could not. In clinical practice, intraoperative core body temperature of >36.0°C seems safer for patients. A recent retrospective study reported that when intraoperative body temperature was >35.4°C, each 0.5°C increase was associated with serious infectious complications (16). In our study, when hyperthermia (>37.3°C) occurred, the increasing duration and AUC predicted PPI and SSI. This is the first study that focused on the relationship between hyperthermia and infectious complications. It suggests that we should foucs our attention on the consequences and prevention of hyperthemia other than hypothemia.

The Centers for Disease Control and Prevention (CDC) of the USA recommends maintaining normal body temperature during surgery to reduce SSI development (15), but no consensus has been reached regarding the normal body temperature range. Normal body temperature is usually 37.0°C (17). A recent study, which analyzed more than 35,000 patients, found the mean oral temperature to be 36.6°C, with a 95% range of 35.7–37.3°C (13). Therefore, in our study, a body temperature of <36.0°C was defined as hypothermia, a body temperature of >37.3°C was defined as hyperthermia, and 36.0–37.3°C was set as the normal body temperature range.

It is known that hypothermia impairs immune function and also induces subcutaneous vasoconstriction and tissue hypoxia, which can disrupt neutrophil function (18, 19). All these mechanisms might contribute to the development of PPI and SSI. Hyperthermia or fever is a normal response to fight infection, and patients with infection who are unable to mount a hyperthermia response will experience an increased risk of mortality (20). It was reported that hyperthermia could increase the phagocytic activity of leukocytes and the mobility of leukocytes, which enhances innate immunity (21). However, in our study, the increasing duration or AUC of hyperthermia was associated with PPI or SSI. In the pneumonia model of animal studies, it was observed that hyperthermia greatly increased neutrophil infiltration and eliminated viable pathogens, but hyperthermia could also cause the loss of endothelial and epithelial barrier function and epithelial injury of the lungs through various pathways (22). In addition, hyperthermia could aggravate oxygen-induced toxicity in the lungs when a high oxygen concentration is applied during anesthesia (23). Our study indicated that hypothermia and hyperthermia could increase the risk of PPI and SSI with a longer duration or a larger AUC. Hence, body temperature should be monitored routinely during surgery to avoid hypothermia and hyperthermia to reduce the risk of PPI and SSI. Unfortunately, only 10.7% of surgical patients in China receive active temperature monitoring during surgery (24), which might be even less in other developing countries. Therefore, more focus should be aimed at improving temperature monitoring and avoiding hypothermia and hyperthermia using active measures.

In this study, patients with PPI or SSI were more likely to develop hypothermia or hyperthermia, which was an independent risk factor for PPI or SSI, although the temperature differences were clinically less significant. It was observed that there were differences in the preoperative ALB levels between the cohort with and without PPI or SSI, although they were at a normal level (>35 g/L). The preoperative ALB level was also an independent risk factor for PPI but not for SSI. The relationship between the preoperative ALB level and postoperative infection needs to be elucidated. In this study, surgical duration was another independent risk factor for PPI and SSI, and patients with PPI or SSI were more likely to experience a longer surgical duration (Supplementary Tables 3–5). Therefore, it is necessary to perform surgical procedures as quickly as possible to reduce postoperative infections.

There are some limitations in our study. First, this study is a single-center retrospective study. Although intraoperative hypothermia or hyperthermia could predict PPI or SSI, we should be cautious when extrapolating the results of the study to other medical centers. Second, we placed the temperature probe in the oropharynx or nasopharynx to monitor core body temperature. The criteria for normal temperature and hyperthermia have not come to a consensus. We used the word “hyperthermia,” which refers to body temperature above the normal value, and defined it as above 37.3°C. Many studies have set normal body temperature at >36.0°C (25), 36.0–37.5°C (26), 36.5–37.0°C (10), or 37.0°C (17). Therefore, with different criteria for hyperthermia, the results of the relationship between hyperthermia and infections might be different. Third, in our study, only 269 patients experienced both hypothermia and hyperthermia during surgery. Although no interaction was observed between hyperthermia and hypothermia in the absolute value, duration, and AUC, we did not categorize the indices into quartiles for the interaction analysis due to the small sample size. Further analysis should be conducted in the future.

In conclusion, the duration and area under the curve of intraoperative hypothermia (<36.0°C) and hyperthermia (>37.3°C) can predict postoperative pulmonary infection and surgical site infection in major non-cardiac surgery, but the absolute value of hypothermia and hyperthermia cannot. The focus of future studies should be aimed at improving the monitoring of intraoperative body temperature to avoid hypothermia and hyperthermia.

## Data availability statement

The original contributions presented in the study are included in the article/Supplementary material, further inquiries can be directed to the corresponding author.

## Ethics statement

The studies involving humans were approved by the Ethics Committee of Chongqing University Cancer Hospital (No. CZLS2024020-A). The studies were conducted in accordance with the local legislation and institutional requirements. Written informed consent for participation was not required from the participants or the participants' legal guardians/next of kin in accordance with the national legislation and institutional requirements.

## Author contributions

Q-YP: Project administration, Writing – original draft, Software, Formal analysis. Y-JY: Writing – original draft, Validation, Software, Data curation. Y-MF: Writing – original draft, Validation, Data curation. S-FS: Writing – original draft, Validation, Data curation. H-LL: Project administration, Writing – review & editing, Writing – original draft, Supervision, Funding acquisition, Conceptualization.
