# Supplementary material for: Relationship between intraoperative hypothermia and hyperthermia with postoperative pulmonary infection and surgical site infection in major non-cardiac surgery
Source: Front Med (Lausanne). 2024 Aug 12;11:1408342. doi: 10.3389/fmed.2024.1408342 (PMC11345182; doi:10.3389/fmed.2024.1408342)
Supplement: Supplementary file 1 [file Table_1.DOCX]

Supplemental Table 1 The characteristics of patients with and without admission to ICU after surgery

| Variable | ICU admission | Non-ICU admission | P |
| --- | --- | --- | --- |
| Age(y) | 53(46,63) | 53(46,63) | 0.339 |
| Gender |  |  | 0.490 |
| Male | 1841(34.76%) | 11000(34.28%) |  |
| Female | 3455(65.24%) | 21092(65.72%) |  |
| BMI (kg/cm^2^) | 23.6(21.5,25.8) | 23.4(21.4,25.7) |  |
| ASA PS |  |  | 0.105 |
| I | 57(1.08%) | 325(1.01%) |  |
| II | 3017(56.97%) | 18581(57.9%) |  |
| III | 2141(40.43%) | 12827(39.97%) |  |
| IV | 79(1.49%) | 349(1.09%) |  |
| V | 2(0.04%) | 10(0.03%5) |  |
| Comorbidity |  |  |  |
| Heart disease | 102(1.93%) | 701(2.18%) | 0.230 |
| Hypertension | 858(16.2%) | 5332(16.61%) | 0.453 |
| DM | 367(6.93%) | 2099(6.54%) | 0.290 |
| Renal disease | 15(0.28%) | 112(0.35%) | 0.446 |
| Liver disease | 130(2.45%) | 726(2.26%) | 0.386 |
| Arrhythmia | 284(5.36%) | 1718(5.35%) | 0.978 |
| Stroke | 100(1.89%) | 528(1.65%) | 0.202 |
| COPD | 24(0.45%) | 153(0.48%) | 0.817 |
| Thyroid disease | 81(1.53%) | 442(1.38%) | 0.382 |
| Smoking | 699(13.20%) | 4104(12.84%) | 0.408 |
| Preoperative medication |  |  |  |
| Antihypertension | 437(8.25%) | 2570(8.01%) | 0.546 |
| Antiplatelets | 48(0.91%) | 271(0.84%) | 0.650 |
| Anticoagulants | 9(0.17%) | 42(0.13%) | 0.475 |
| Hypoglycemic | 237(4.48%) | 1336(4.16%) | 0.295 |
| Preoperative tests |  |  |  |
| Hb(g/L) | 127(121,136) | 127(121,137) | 0.329 |
| Glucose(mmol/L) | 5.07(4.48,5.22) | 5.02(4.48,5.27) | 0.763 |
| ALB(g/L) | 42(39.9,44.07) | 42(39.7,44.35) | 0.971 |
| WBC (*10^9/L) | 6.15(4.99,6.58) | 6.15(4.98,6.70) | 0.562 |
| Surgical duration(min) | 136(95,210) | 135(95,205) | 0.238 |
| Bleeding (ml) | 100(50,100) | 100(50,100) | 0.801 |
| Transfusion | 113(2.13%) | 607(1.89%) | 0.235 |
| Fluid infusion(ml) | 1600(1200,2400) | 1600(1200,2300) | 0.000 |
| Hypothermia | 869(16.41%) | 5304(16.53%) | 0.829 |
| Hyperthermia | 199(3.76%) | 1185(3.69%) | 0.816 |

BMI: body mass index; ASA PS: american society of anesthesiologists classification physical status; DM: diabetes mellitus; COPD: chronic obstructive pulmonary disease; Hb: hemoglobin; ALB: albumin; WBC: white blood cell.
