# Supplementary material for: Relationship between intraoperative hypothermia and hyperthermia with postoperative pulmonary infection and surgical site infection in major non-cardiac surgery
Source: Front Med (Lausanne). 2024 Aug 12;11:1408342. doi: 10.3389/fmed.2024.1408342 (PMC11345182; doi:10.3389/fmed.2024.1408342)
Supplement: Supplementary file 2 [file Table_2.DOCX]

Supplemental Table 2 The odds of PPI and SSI in patients without admission to ICU after surgery

|  | PPI | | SSI | |
| --- | --- | --- | --- | --- |
|  | aOR (95%CI) | *P* value | aOR (95%CI) | *P* value |
| Hypothermia |  |  |  |  |
| Nadir value(^0^C)  Q1(<35.3) (n=335)  Q2(35.3-35.7) (n=1274)  Q3(35.7-35.9) (n=1867)  Q4(≥35.9) (n=1446)  Duration(min) | 0.699(0.359,1.360)  0.930(0.673,1.284)  0.811(0.611,1.078)  1.007 (0.751,1.350) | 0.291  0.659  0.150  0.962 | 0.688 (0.219,2.155)  0.623 (0.320,1.213)  0.878 (0.551,1.399)  0.969 (0.585,1.607) | 0.521  0.164  0.584  0.904 |
| Q1(0-30) (n=1510) | 0.821(0.599,1.124) | 0.218 | 0.992(0.607,1.621) | 0.973 |
| Q2(30-90) (n=1799) | 0.940 (0.718,1.232) | 0.656 | 0.720(0.428,1.211) | 0.215 |
| Q3( 90-195) (n=1321) | 0.935(0.680,1.287) | 0.682 | 0.740(0.404,1.355) | 0.329 |
| Q4(>195) (n=292) | 0.781(0.385,1.584) | 0.493 | 1.196(0.442,3.233) | 0.724 |
| AUC(^0^C _*_ min) |  |  |  |  |
| Q1(<1077) (n=1170) | 0.810 (0.566,1.159) | 0.249 | 0.594 (0.294,1.202) | 0.147 |
| Q2(1077-3198) (n=1807) | 0.925 (0.705,1.215) | 0.577 | 1.068(0.691,1.651) | 0.766 |
| Q3(3198-6946) (n=1598) | 0.955 (0.716,1.273) | 0.752 | 0.608 (0.332,1.112) | 0.106 |
| Q4(>6946) (n=347) | 0.726 (0.372,1.414) | 0.346 | 1.483 (0.656,3.356) | 0.344 |
| Hyperthermia  Peak value(^0^C)  Q1(=37.3) (n=270)  Q2(37.3-37.5) (n=300)  Q3(37.5-38) (n=215)  Q4(>38) (n=56) | 1.799(1.064,3.043)  1.069(0.567,2.016)  1.166(0.573,2.373)  1.772(0.551,5.593) | 0.028  0.837  0.671  0.337 | 0.675(0.167,2.726)  1.219(0.451,3.299)  1(omitted)  1(omitted) | 0.581  0.696 |
| Duration(min) |  |  |  |  |
| Q1(0-15) (n=146) | 1.539(0.718,3.298) | 0.268 | 1(omitted) |  |
| Q2(15-75) (n=380) | 1.262(0.750,2.125) | 0.381 | 1.204(0.494,2.930) | 0.683 |
| Q3(75-210) (n=274) | 1.530(0.872,2.683) | 0.138 | 0.332(0.046,2.370) | 0.271 |
| Q4(>210) (n=41) | 0.783(0.107,5.730) | 0.810 | 1(omitted) |  |
| AUC(^0^C _*_ min) |  |  |  |  |
| Q1(<560) (n=115) | 1.676(0.735,3.824) | 0.220 | 1(omitted) |  |
| Q2(560-2803) (n=374) | 1.190(0.695,2.034) | 0.526 | 1.220(0.501,2.670) | 0.661 |
| Q3(2803-7945) (n=309) | 1.685(1.013,2.801) | 0.044 | 0.294(0.041,2.102) | 0.223 |
| Q4(>7945) (n=43) | 1(omitted) |  | 1(omitted) |  |

SSI: surgical site infection; PPI: postoperative pulmonary infection; Q1: the 1st quartile; Q2: the 2nd quartile; Q3: the 3rd quartile; Q4: the 4th quartile. Nadir value: the lowest value under hypothermia; Peak value: the highest value under hyperthermia; AUC: area under the curve.
