# Supplementary material for: Relationship between intraoperative hypothermia and hyperthermia with postoperative pulmonary infection and surgical site infection in major non-cardiac surgery
Source: Front Med (Lausanne). 2024 Aug 12;11:1408342. doi: 10.3389/fmed.2024.1408342 (PMC11345182; doi:10.3389/fmed.2024.1408342)
Supplement: Supplementary file 3 [file Table_3.DOCX]

Supplemental Table 3 Independent risk factors for PPI from multivariate logistic analysis

| Variable | OR | 95%CI | *P* value |
| --- | --- | --- | --- |
| Age (years old) | 1.023 | 1.018, 1.029 | 0.000 |
| Sex (Male vs Female) | 1.840 | 1.611, 2.101 | 0.000 |
| BMI (kg/m^2^) | 0.981 | 0.964, 0.999 | 0.036 |
| ASA (III-V vs I-II) | 1.922 | 1.672, 2.210 | 0.000 |
| ALB (g/L) | 0.967 | 0.954,0.980 | 0.000 |
| Surgical duration(min) | 1.002 | 1.002, 1.003 | 0.000 |
| Hypothermia (Yes vs No) | 1.392 | 1.212, 1.608 | 0.000 |

Supplemental Table 4 Independent risk factors for SSI from multivariate logistic analysis

| Variable | OR | 95%CI | *P* value |
| --- | --- | --- | --- |
| Age (years old) | 1.014 | 1.005, 1.024 | 0.002 |
| Sex (Male vs Female) | 1.949 | 1.536, 2.474 | 0.000 |
| ASA (III-V vs I-II) | 1.303 | 1.029, 1.649 | 0.028 |
| Hb (g/L) | 0.985 | 0.979,0.991 | 0.000 |
| Surgical duration(min) | 1.004 | 1.003, 1.005 | 0.000 |
| Hypothermia (Yes vs No) | 1.356 | 1.052, 1.748 | 0.023 |
| Hyperthemia (Yes vs No) | 1.581 | 1.038, 2.399 | 0.025 |

Supplemental Table 5 Surgical duration in different surgical types

| Surgical type | Surgical duration (min) |
| --- | --- |
| Breast surgery | 125(100,157) |
| Head and neck surgery | 100(76,142.5) |
| Gynecological surgery | 130(95,189) |
| Urology surgery | 155(97.5,251) |
| Neurosurgery | 230(157,314.5) |
| Hepatobiliary surgery | 195(108,325) |
| Gastrointestinal surgery | 200(136,260) |
| Thoracic surgery | 140(105,195) |
| Orthopedic surgery | 115(83,166) |
| Others | 115.5(85,179.5) |

Surgical duration was presented as median (interquartile range).
